# TRPV1: A Target for Rational Drug Design

**DOI:** 10.3390/ph9030052

**Published:** 2016-08-23

**Authors:** Vincenzo Carnevale, Tibor Rohacs

**Affiliations:** 1 Institute for Computational Molecular Science, Temple University, Philadelphia, PA 19122, USA; 2 New Jersey Medical School, Rutgers University, Newark, NJ 07103, USA

**Keywords:** TRPV1, capsaicin, vanilloid, pain, nociception

## Abstract

Transient Receptor Potential Vanilloid 1 (TRPV1) is a non-selective, Ca^2+^ permeable cation channel activated by noxious heat, and chemical ligands, such as capsaicin and resiniferatoxin (RTX). Many compounds have been developed that either activate or inhibit TRPV1, but none of them are in routine clinical practice. This review will discuss the rationale for antagonists and agonists of TRPV1 for pain relief and other conditions, and strategies to develop new, better drugs to target this ion channel, using the newly available high-resolution structures.

## 1. Introduction—TRP Channels

Transient Receptor Potential (TRP) channels were discovered while analyzing a visual mutant of drosophila that showed transient response to light, as opposed to the sustained receptor potential in wild type flies [1]. Unlike in mammals, invertebrate vision is initiated by the activation of a Phospholipase C (PLC) enzyme, which hydrolyzes the membrane phospholipid phosphatidylinositol 4,5-bisphosphate (PIP_2_) to form the two classical second messengers inositol 1,4,5 trisphosphate (IP_3_) and Diacylglycerol (DAG). Despite decades of research, it is still unclear how this enzymatic cascade activates the channel responsible for generating the receptor potential in insects. This channel complex includes dTRP protein, mutation of which was responsible for the transient light response [2]. Based on sequence homology, mammalian orthologues of the dTRP channel were soon cloned; the seven mammalian TRPs with the closest homology to dTRP were designated as Classical, or Canonical TRPs, or TRPCs [3]. Two additional major subfamilies (TRPV and TRPM) and three smaller subfamilies (TRPA, TRPN, and TRPML) were identified; together with TRPCs they comprise the mammalian TRP (super) family. TRP channels are highly diverse; it is impossible to succinctly summarize their functions. Two major general themes however stand out: regulation by the PLC pathway, and involvement in sensory transduction. The closest mammalian homologs of the dTRP channel, TRPCs are all stimulated downstream of PLC coupled receptor activation, and several other TRP channels are modulated by this pathway [4]. TRP channels are involved in a variety of sensory functions; their roles are best established in thermosensation [5]. Mutations in TRP channels cause human diseases as diverse as kidney disease (TRPC6), spontaneous pain syndrome (TRPA1), hypomagnesemia (TRPM6), night blindness (TRPM1) and complex musculoskeletal and neurological disorders (TRPV4) [6]. Given their widespread physiological roles and relatively recent discovery, many of them are promising drug targets [7].

## 2. Sensory TRP Channels

TRP channels play various roles in all primary senses [8]. They initiate the visual signal in invertebrates, and TRPM1 in retinal on-bipolar cells plays an important role in visual transmission in mammals; its loss of function mutation causes stationary night blindness in humans [9]. TRPM5 knockout mice have altered sweet, bitter and umami taste sensation [10]. TRP channels play important roles in mechanosensation in invertebrates, but their role in mammals is controversial [11]. TRPC2 is important in pheromone sensation in rodents, but in humans it is a pseudogene [8]. As discussed below, the roles of TRP channels are best established in thermosensation, and chemical nociception [5,12]. To place TRPV1 channels in context, we first briefly discuss thermo- and somatosensory TRP channels other than TRPV1.

### 2.1. Heat Sensitive TRP Channels other than TRPV1

TRPV2 [13] is a capsaicin insensitive homolog of TRPV1, initially identified as a noxious heat sensor. It is activated with a heat threshold higher than that for TRPV1, and it is well expressed in peripheral sensory dorsal root ganglion (DRG) neurons [14]. Behavioral studies however found no difference in temperature sensation between TRPV2^−/−^ and wild type mice, showing that this channel is unlikely to be a physiological heat sensor [15]. TRPV2 knockout mice show various abnormalities, such as macrophage phagocytosis [16], and maintenance of cardiac structure and function [17], highlighting the importance of these channels in functions other than thermosensation.

TRPV3 [18] is expressed in keratinocytes of the skin and it is activated by moderate heat [19]. These channels are sensitized and activated by various oregano, thyme and clove derived skin sensitizers, such as carvacrol, thymol and eugenol. While some studies reported defects in temperature sensation in TRPV3^−/−^ mice, the effect depended on the genetic background [5]. In humans, gain of function mutations of TRPV3 lead to Olmsted syndrome, which is characterized by palmoplantar and periorificial keratoderma, alopecia and severe itching [20].

TRPV4 [21] is an osmosensor, but it is also activated by moderate heat. Similar to TRPV3, it is essentially undetectable in DRG neurons, but well expressed in keratinocytes [5]. TRPV4 knockout mice showed a mild defect in thermal preference [22].

TRPM3 is the most recent addition to the thermo-TRP family. These channels are expressed in small nociceptive DRG neurons; they are activated by heat, and chemical agonists such as pregnenolone sulfate [23]. Genetic deletion of these channels in mice leads to defects in noxious heat sensitivity [24].

### 2.2. Cold Sensitive TRP Channels

TRPM8 [25] is a well-established sensor of mild environmental cold temperatures. This channel is activated by cold, menthol, and other cooling agents, such as icilin [19,26,27]. Genetic deletion of these channels in mice leads to decreased sensitivity to moderate cold [28,29,30]. TRPM8 is also the main mediator of menthol-induced analgesia [31].

TRPA1 [32] was originally proposed to function as a noxious cold sensor [33]. This channel is also activated by a variety of noxious and pungent chemical compounds such as mustard oil and acrolein [34]. While it is very well established by now that this channel serves as a noxious chemical sensor, its cold activation is still controversial, detailed discussion of this controversy is beyond the scope of this chapter, and it was reviewed in detail recently [5,32].

TRPC5 channels [35], but not TRPC1/TRPC5 heteromers, were shown to be activated by cold temperatures, but genetic deletion of TRPC5 did not result in a change in cold sensitivity [36].

## 3. Physiological and Pathophysiological Roles of TRPV1

Similarly to most TRP channels, TRPV1 is an outwardly rectifying Ca^2+^ permeable non-selective cation channel. Its activators most often used in experiments are temperatures over 42 °C, capsaicin, and low extracellular pH. Resiniferatoxin (RTX) is an ultrapotent agonist of TRPV1 [37], it activates the channel at lower concentrations than capsaicin, but its effect also develops much slower [38]. Most likely due to its slowly developing effect [39], RTX is less pungent than capsaicin [40].

TRPV1 is one of the most promiscuous ion channels; it is activated by many endogenous [41] and exogenous compounds, including various painful arthropod toxins [42,43]. Endogenous regulators of TRPV1 include endovanilloids, such as anandamide and 2-Arachidonoylglycerol [44] and lysophosphatidic acid [45]. Most of these endogenous compounds, even at maximally effective concentrations, induce TRPV1 currents smaller than those evoked by saturating capsaicin concentrations. Interestingly, ethanol at high concentrations also activates TRPV1, an effect probably responsible for the pungency of concentrated spirits [46].

TRPV1 has been implicated in a plethora of physiological and pathophysiological functions, which have been extensively reviewed [47,48]. Below we will summarize the functions most relevant to its role as a drug target.

### 3.1. Functions Related to Expression in Peripheral Sensory Neurons

TRPV1 was first described in small nociceptive DRG and trigeminal ganglion (TG) neurons. Most sensory neurons innervating the urinary bladder also express TRPV1. This channel is also present in a large portion of neurons of the nodose and jugular sensory vagal ganglia [49] innervating the airways (see Section 3.1.3). The following three sections will briefly discuss the roles of TRPV1 in nociception, micturition, and airway hypersensitivity.

#### 3.1.1. Nociceptive Heat Sensation and Thermal Hyperalgesia

Capsaicin, the chemical activator of TRPV1 evokes intense burning pain, and temperatures over ~42 °C induce a steep increase in channel activity with a ~20 fold change upon a 10 °C increase in temperature (Q_10_ ~20) [50]. These properties intuitively suggest that this channel functions as a noxious heat sensor. Surprisingly, initial studies in mice somewhat differed on the effect of genetic deletion of this channel on acute nociceptive heat sensation [51]. One knockout study found that TRPV1^−/−^ mice showed altered responses to heat in the tail flick, radiant heat, and hot plate assays [52]. An independent study however found no difference in acute heat sensitivity between wild type and TRPV1^−/−^ mice [53]. The same study, on the other hand, found that thermal hyperalgesia induced by inflammation was severely altered in the TRPV1 knockout mice [53]. Subsequent research with TRPV1^−/−^ mice supported the role of TRPV1 as a noxious heat sensor having more pronounced defects at higher temperatures [15,54,55]. Also consistent with the role of TRPV1 as a noxious heat sensor is the finding that one of the key side effects of TRPV1 blockers in humans is the increase of the threshold for painful heat, and consequential accidental burns (see Section 4.1) [7].

When TRPV1 expressing sensory neurons were ablated using diphtheria toxin in mice, heat sensitivity was essentially eliminated [56]. The more robust phenotype of this mouse model compared to TRPV1^−/−^ mice suggests the presence of other heat sensing ion channels in TRPV1 expressing neurons.

Thermal hyperalgesia, increased sensitivity to heat, is a hallmark of inflammatory pain. In mouse models, genetic deletion of TRPV1 essentially eliminated thermal hyperalgesia induced by inflammation in two independent TRPV1^−/−^ lines [52,53], demonstrating the importance of these channels in this phenomenon. In principle, there are two mechanisms by which TRPV1 activity can increase and thus induce thermal hypersensitivity: higher expression levels, and increased sensitivity/activity. Increased expression of TRPV1 at the RNA and protein levels has been reported both in inflammatory models [57], and in response to application of inflammatory mediators such as Nerve Growth Factor (NGF) [58]. In addition, NGF was shown to increase the number of functional TRPV1 channels in the plasma membrane by PI3K mediated increase in trafficking [59,60]. In contrast to NGF, stimulation of pro-inflammatory G-protein coupled receptors (GPCRs) increase the sensitivity of TRPV1 to heat, low pH and capsaicin, without increasing the number of functional channels [61]. Direct phosphorylation by Protein Kinase C (PKC) has been shown to play an important role in sensitization of TRPV1 by pro-inflammatory mediators such as bradykinin, prostaglandins and extracellular ATP, all of which act via Gq-coupled receptors [62,63,64]. Some pro-inflammatory mediators, such as prostaglandin E2 also activate Gs-coupled receptors, and the ensuing cAMP formation and activation of Protein Kinase A (PKA) contributes to sensitization [64,65,66]. The A kinase anchoring protein 79/150 (AKAP79/150) was shown to be important not only to PKA-, but also for PKC-mediated potentiation of TRPV1 activity [64], highlighting the importance of localized signaling complexes in the regulation of these channels.

In contrast to the very well established role of TRPV1 in inflammatory thermal hyperalgesia, the role of TRPV1 in rodent models of neuropathic pain is less clear. Caterina et al. reported no difference between wild type and TRPV1^−/−^ mice in thermal hyperalgesia induced by partial sciatic nerve ligation [52]. This is consistent with the finding that in rodent models of neuropathic pain TRPV1 expression is often decreased rather than increased [67], even though in some nerve injury models increased TRPV1 expression was also reported [68].

#### 3.1.2. Urinary Bladder

The majority of sensory nerves innervating the urinary bladder express TRPV1, but the expression of this channel in urothelial cells is controversial; if present, it is expressed at much lower levels than in sensory neurons [7]. The role of TRPV1 in normal micturition is not very clear; TRPV1^−/−^ mice display a only mild spotty incontinence [69], and TRPV1 antagonists do not alter micturition in naïve mice [7]. The role of TRPV1 on the other hand is very well established in dysfunctional micturition reflex. Desensitizing TRPV1 expressing nerves using intravesical capsaicin, or RTX have been used to treat incontinence [7]. RTX is better tolerated, because it causes less pain than capsaicin.

#### 3.1.3. Airways

TRPV1 is expressed in neurons of the vagal nerve innervating the airways that give rise to C-fibers. The cell bodies of these neurons are found in the nodose and jugular ganglia. Inhaled capsaicin evokes an urge to cough in humans and guinea pigs, but not in rats [7]. Sensitivity to inhaled capsaicin aerosols is increased in some respiratory disorders, and chronic allergic airway inflammation leads to increased TRPV1 expression [49]. TRPV1 antagonists were reported to inhibit cough evoked by inhaled aerosoled citric acid, but not spontaneous cough [7]. Desensitizing TRPV1 by intranasal application of capsaicin provided symptomatic relief in vasomotor rhinitis, but the procedure was painful and not very well tolerated [70]. Sensory neurons innervating the airways also express TRPA1, consistent with the role of that channel as a major sensor of environmental irritants [71].

### 3.2. Functions Based on Expression in Other Tissues

There is a relatively large literature on the involvement of TRPV1 in many different functions in a variety of tissues, especially in the central nervous system. Some effects are well established, some of the data are disputed, mainly due to findings obtained from a reporter mouse, where the authors show very restricted expression pattern of TRPV1 in small peptidergic neurons of primary sensory ganglia such as DRG and TG, the caudal hypothalamus, and in a subset of arteriolar smooth muscle cells within thermoregulatory tissues [72]. Below we briefly discuss three topics: 1. body temperature regulation, which is important for understanding the side effects of TRPV1 antagonists, 2. increased metabolism, which is a potential positive effect of TRPV1 agonists 3. the potential role of TRPV1 in ocular injury, which may also be a potential therapeutic application for TRPV1 antagonists.

#### 3.2.1. Body Temperature Regulation

Given that TRPV1 does not normally show significant activity below ~42 °C, it is counterintuitive that this channel plays a major role in regulation of body temperature. Furthermore, TRPV1^−/−^ mice have basal body temperatures indistinguishable from wild-type mice, even though they do not show the characteristic drop in body temperature after capsaicin injection [52]. Mice, in which TRPV1 expressing cells are depleted, also show no difference from wild-type animals in basal body temperature, but they show somewhat altered ability to maintain their body temperatures in response to various challenges [56]. Some investigators concluded that TRPV1 is not a major regulator of body temperature [73].

A major side effect of TRPV1 antagonist of TRPV1, however, is hyperthermia, which in some cases may reach dangerous levels [7]. How is this possible? As not all antagonists have this effect, it is unclear at this point whether this is an on target side effect [7]. It also has to be kept in mind that the temperature threshold of TRPV1 is dynamic; inflammatory mediators for example shift the temperature threshold in a PKC dependent manner. It is quite possible, that TRPV1 channels expressed in cells responsible for body temperature regulation, are in a sensitized state. The role of TRPV1 in thermoregulation has been recently reviewed in detail [74].

#### 3.2.2. Metabolism

There is extensive literature on the effects of capsaicin and TRPV1 on metabolism, diabetes and obesity, as reviewed in [7,75]. A relatively recent meta-analysis of clinical trials concluded that daily consumption of capsaicin or capsiate, a non-pungent vanilloid, modestly increase thermogenesis and decrease appetite, thus can be useful in weight management [76]. Animal studies however show a somewhat more confusing picture. TRPV1^−/−^ mice were shown to be protected from diet-induced obesity [77], but a more recent study found no difference in weight gain on high fat diet between wild type and TRPV1 knockout mice [78]. Neonatal ablation of TRPV1 expressing neurons using capsaicin in rats also resulted in conflicting data: some authors found lower weight, while others found no change in food consumption (reviewed in [79]). Consistent with the human data on the other hand, capsaicin consumption was shown to induce browning of white adipose tissue and reduce obesity in mice in a TRPV1 dependent fashion [80].

#### 3.2.3. Ocular Injury and Eye Disease

Besides their potential use as analgesics, TRPV1 antagonists may also be useful in other pathological conditions [7], one example; eye injury is briefly discussed here. Okada et al. showed that in an alkali burn model of cornea in mice, both genetic deletion of TRPV1, and TRPV1 antagonists inhibited inflammatory cell invasion, myofibroblast generation, and scarring [81]. It was also shown that TRPV1 activation induces EGFR-transactivation in human corneal epithelial cells, which increases cell proliferation [82]. It was also demonstrated that TRPV1 activation in corneal epithelial cells by hypertonic media, similar to those observed in tears of dry eye patients, induces increased pro-inflammatory and chemoattractant release, which may contribute to the development of inflammation in dry eye patients [83]. The role of TRP channels in ocular function and eye disease has been recently reviewed [84].

## 4. TRPV1 as a Drug Target

There are many different TRPV1 antagonists developed by the pharmaceutical industry, several of them are, or were, in clinical trials, but to our knowledge, none of them are in routine clinical use yet. The topic has been extensively reviewed recently [7,85], here we will only discuss two aspects briefly: the limitations of currently available TRPV1 antagonists, and the rationale for using TRPV1 agonists for pain relief.

### 4.1. Limitations of TRPV1 Antagonists

Given the importance of TRPV1 in nociception, the rationale for using its antagonists as pain medications is straightforward. TRPV1 antagonists have been shown to provide pain relief in some pain modalities in animal models, but they are not universally effective in all studies. This topic has been extensively reviewed [86,87,88], here we only mention a small number of examples. In a rat model of osteoarthritis the intraarticular injection of the TRPV1 antagonist JNJ17203212 almost completely eliminated the weight bearing asymmetry in mice [89]. A different TRPV1 antagonist ABT-116, however induced only moderate pain relief in dogs in an experimental model of synovitis [90], and a third TRPV1 antagonist AZD1386 was withdrawn from clinical trials because of the absence of significant clinical benefit in osteoarthritis patients [91]. TRPV1 antagonists inhibited acute pancreatitis-induced pain in rats, but they were ineffective once chronic pancreatitis was established [92]. In mice the TRPV1 antagonist JNJ17203212 was shown to inhibit bone cancer pain [93].

Besides their variable and modality dependent effectiveness, the major limitation of TRPV1 antagonists is the two key side effects: accidental burns and hyperthermia. While accidental burns are usually mild and can be largely avoided by warning the patients to be careful, hyperthermia in some cases can be severe [7]. Is it possible to develop antagonists that are devoid of these side effects? Different antagonists show these side effects to different extents, suggesting that the answer is yes. Accumulating knowledge about the regulation of the channel also raises the possibility of developing better antagonist.

TRPV1 is a multimodal ion channel, the regulation of which is complex. Heat, low pH and capsaicin activate the channel with different mechanisms, thus theoretically it is possible to develop modality dependent inhibitors. For example, an antagonist that does not block heat activation of the channel, is expected not to block nociceptive heat sensation, thus should not cause accidental burns. Such an inhibitor may still have beneficial effects by inhibiting other modalities, such as activation by low pH and by endovanilloids. An inhibitor that selectively blocks the sensitized state of the channel, but not the basal activity, could potentially be also devoid of this side effect. What makes this rationale a little more complex is that it is not very well known what activates these channels in various pain conditions. In addition, it is not currently clear whether the most important side effect, hyperthermia, is an on-target, or off-target effect [7]. One could argue that inhibiting a heat-sensing channel is expected to induce an increase in body temperature, by “tricking” the body into feeling colder, thus increasing temperature to compensate. There are two problems with this thought: first, as mentioned earlier, the temperature threshold of TRPV1 is ~42 °C, well over normal body temperature. Second, it was proposed that antagonists that do not block activation of the channel by low pH are less likely to induce hyperthermia [94]. This, however, seems to be true for some antagonists, but not for others [7].

### 4.2. Desensitization of TRPV1–Rationale for Using TRPV1 Agonists

Capsaicin containing creams and other topical preparations have long been used as local analgesics. Topical capsaicin, after an initial burning sensation, desensitizes the sensory nerves not only to heat and capsaicin, but other modalities as well [79]. Capsaicin-induced desensitization is a poorly defined and highly complex phenomenon.

Native TRPV1 in DRG neurons and heterologously expressed TRPV1 undergo desensitization when exposed to maximally effective concentrations of capsaicin [50,95], which is defined as decreased current amplitude in the continuous presence of the agonist. Some articles differentiate between acute desensitization, and tachyphylaxis, which is reduced responsiveness to short applications of capsaicin. The best-established mechanism behind desensitization is depletion of PIP_2_ by Ca^2+^-induced activation of PLC. Even though PIP_2_ was originally proposed to inhibit TRPV1 [96], it is clear by now that the channel is potentiated by this lipid applied to excised inside-out patches [59,97,98,99,100], and the channel requires endogenous phosphoinositides for activity in a cellular context. Capsaicin was shown to activate a PLCδ isoform leading to a robust decrease in the levels of PIP_2_ and its precursor PI(4)P in TRPV1 positive DRG neurons [101]. Various inducible phosphoinositide phosphatases that decrease PIP_2_ and PI(4)P levels were shown to inhibit TRPV1 activity [97,101,102,103], and the decrease in PIP_2_ levels displayed very good correlation with decreasing current levels [102]. When the whole cell patch pipette is supplemented with PIP_2_, or PI(4)P, desensitization of both recombinant TRPV1 [99,104,105] and native TRPV1 in DRG neurons [98] is reduced, but not eliminated, pointing to the contribution of other signaling processes. Paradoxically, the decrease in PIP_2_ levels upon PLC activation by GPCRs may also play an auxiliary role in sensitization of TRPV1 by potentiating the effect of PKC [101]. Phosphoinositide regulation of TRPV1 is complex, its detailed discussion is beyond the scope of this article, and was extensively reviewed recently [106].

Other Ca^2+^ dependent processes such as calmodulin (CaM) [107], calcineurin [108,109], and endocytosis [110] may also contribute to desensitization. The distal C-terminus of TRPV1 was shown to bind CaM, and removal of this segment reduced tachyphylaxis, and to a lesser also inhibited acute desensitization [107]. The same study however found that neither the CaM inhibitor W7, nor a dominant negative mutant of CaM inhibited desensitization [107]. An independent study found that CaM applied to excised patches inhibited TRPV1 activity, this inhibition, however was slow, developing over several minutes, and partial [111]. An anti-CaM antibody was also shown to inhibit desensitization [104], and the crystal structure of CaM binding to the distal C-terminus of TRPV1 has been determined [112]. Inhibition of the Ca^2+^-CaM dependent protein phosphatase calcineurin with cyclosporine and intracellular dialysis of cyclophilin was shown to reduce desensitization of TRPV1 [108,109], but a pseudosubstrate inhibitor of calcineurin failed to inhibit desensitization [113]. Even though current activity was shown to recover from desensitization within ~10 min, if ATP is provided in the patch pipette [114], TRPV1 was also shown to undergo internalization after exposure to capsaicin [110].

TRPV1 activation in DRG neurons does not only lead to desensitization of the TRPV1 channel itself, but the massive Ca^2+^ influx also leads to inhibition of other ion channels via various downstream signaling pathways. There are numerous reports showing that capsaicin application inhibits voltage gated Ca^2+^ channels [115,116,117,118,119,120]. Voltage gated Na^+^ channels were also shown to be partially inhibited upon TRPV1 activation [121]. KCNQ voltage gated K^+^ channels were shown to be inhibited upon TRPV1 activation via PIP_2_ depletion [122]. Hyperpolarization activated HCN channels [123], P2X ATP activated ion channels [124,125] and TRPA1 [126] were also shown to be inhibited by capsaicin via TRPV1 activation. Mechanosensitive Piezo 1 and Piezo2 channels are also inhibited by TRPV1 activation in a Ca^2+^ dependent manner and supplementing the whole cell patch pipette with PIP_2_ alleviated this inhibition [127].

The acute effects of TRPV1 activation on the channel itself and inhibition of other channels may, in principle, contribute to the analgesic effect of topical capsaicin. It has to be noted, however, that the pain relieving effects of high concentrations of topical capsaicin last for several weeks, and local, reversible nerve degeneration has been demonstrated after those treatments [7]. On the other hand, the effects of intravesical RTX treatment for incontinence (see later) are more reversible, and occur without detectable nerve degeneration [7], thus acute inhibition of ion channels is a more feasible explanation for this effect. Note that RTX may cause nerve degeneration when administered via a different route [128].

Even though capsaicin is highly lipophilic, the non-damaged human skin poses a significant barrier to this compound, which is why handling hot chili peppers does not usually cause significant pain in the hands, while capsaicin evokes very intense pain in the mouth, or the eye, when accidentally touched with capsaicin-contaminated hands. The over the counter creams available in US pharmacies usually contain 0.1% capsaicin, which corresponds to ~3.3 mM, which is several orders of magnitude higher concentration than the EC_50_ (~100 nM) when activating the channel in electrophysiological experiments [61]. When placed on human skin, however, these formulations usually evoke only a slowly developing moderate burning sensation, and they require multiple daily applications over several weeks to have an effect [129]. When injected intradermally in a small volume (20 μL), the same, or even lower concentrations of capsaicin (0.01% and 0.1%) were shown to induce a dose-dependent nerve degeneration after a single injection [130]. Overall, capsaicin from sporadic application of low concentration creams may not reach the nerve terminals at high enough concentrations to induce clinically relevant desensitization. It was proposed that these creams are more likely to exert some pain relief via counter irritation [79], which may involve the release of somatostatin, which exerts antinociceptive and anti-inflammatory effects [131,132]. Developing TRPV1 agonists with better penetration through the skin is one area where rational drug design may play a role. It has to be noted however, that when applied in a large quantity and for a long time (48 h), a low concentration (0.1%) capsaicin preparation was also shown to induce long lasting, slowly reversible nerve degeneration [133]. There are several high concentration patches and injectable capsaicin formulations either in clinical trial or approved; these are discussed in detail elsewhere [7]. The high concentration capsaicin patch Qutenza (8%) which requires a single one hour application and its effect lasts for several weeks, for example is approved for post-herpetic neuralgia [129], but did not receive FDA approval for HIV-related pain [7]. Unlike the topical capsaicin creams containing low concentrations of the drug mentioned above, the Qutenza having 8% capsaicin causes degeneration of TRPV1-expressing nociceptive nerve endings even after a 1 h application, which is only slowly reversible, explaining the long duration of action of the drug [134].

The effects of TRPV1 agonists also depend on the application site and age of experimental animals. In newborn rats, systemic injection of RTX, or capsaicin, destroys TRPV1 positive cells, and has been used as a model to study nociception [79,135]. Intrathecal application of RTX or capsaicin induces degeneration of the central terminals of TRPV1 positive DRG neurons, and thus eliminates transmission of signals from these cells [136]. CT-guided local administration of RTX to DRGs in pigs was shown to reduce withdrawal from noxious heat in corresponding dermatomes [137].

### 4.3. Structure Based Rational Design of TRPV1 Modulators

Despite the advances in membrane protein crystallography, and the enormous interest in TRP channels, no full-length TRP channel has been crystallized, until very recently [138]. Advances in cryo-electron microscopy (cryoEM) however made it possible to obtain structures sufficiently accurate to resolve side chain conformations. TRPV1 was the first ion channel for which such structure was available [139,140], followed by TRPA1 [141] and TRPV2 [142,143]. The most recent cryoEM structure of TRPV1 was determined in lipid nanodiscs, a native-like lipid environment [144].

The advances in structural biology of TRP channels opens up the possibility to rationally design both agonists and antagonist for TRPV1 and other TRP channels.

#### 4.3.1. CryoEM Structure of TRPV1: First Insights on the Gating Mechanism

**Figure 1:**
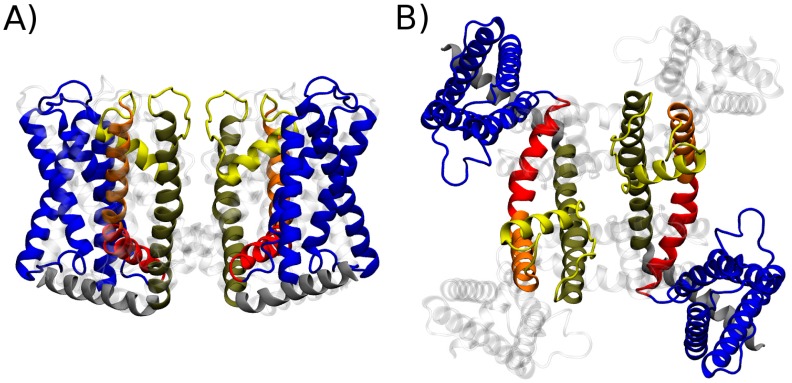
Molecular architecture of the Transient Receptor Potential Vanilloid 1 channel (TRPV1). Cartoon representation of the structure of the transmembrane region of TRPV1 as determined via cryoEM in lipid nanodiscs: (**A**) side view; and (**B**) top view. For clarity, only two subunits are highlighted in solid color while the other two are rendered as grey shading. Different colors highlight the major structural elements described in the text: S1–S4 domain (blue), linker domain (red), S5 (orange), pore helix and selectivity filter (yellow), S6 (brown) and TRP domain (grey).

Facilitated by recent technical developments in single-particle cryoEM, the structure of the mammalian TRPV1 cation channel has been recently solved in three distinct conformational states: closed state without any agonist, capsaicin-bound partially open state, and fully open state with two agonists RTX and double-knot-toxin bound to the channel [139,140]. These structures have provided an unprecedented atomistic view of both the initial and terminal states of channel gating. TRPV1 is a member of the evolutionary superfamily of 6TM channels, i.e., it is distantly related to the family of voltage gated ion channels [145]. The functional assembly of the channel is a tetramer of subunits containing each six transmembrane helices, termed S1 through S6. As a clade of 6TM channels, TRP channels (and thus TRPV1) inherited the overall architecture from voltage sensitive ancestral genes (Figure 1). Thus, besides the pore domain, constituted by the tetrameric assembly of the S5 and S6 helices, TRPV1 shows four identical peripheral domains that are structurally homologous to voltage sensor domains. These are formed by helices S1 through S4 and are connected to the pore domain through an amphiphilic helix parallel to the membrane called S4–S4 linked domain [139].

In contrast to voltage gated ion channels, the S1–S4 domain does not undergo significant conformational changes upon activation. However, in the agonist-activated states of the channel, pore-forming helices move apart from each other, breaking a seal of hydrophobic residues and opening the so-called gate [139]. The molecular mechanism responsible for channel opening upon ligand binding is thus seemingly different from that underlying voltage sensitive activation. In spite of the abundant structural information so far collected, the nature of the close-to-open structural transition and, most importantly, the role played in this transition by the channel-ligand interactions is still the object of intense investigation.

Before the structure of TRPV1 became available, biochemical studies had already localized the interaction sites of various TRPV1 small molecule agonists: capsaicin, RTX, and *N*-arachidonoyl dopamine activate the channel from the intracellular face in a pocket usually referred to as vanilloid binding site [139]. The best-characterized TRPV1 ligand is arguably the agonist capsaicin [79]. Intriguingly, the molecule capsazepine, which is structurally very similar to capsaicin, while showing a reasonably large affinity, does not cause channel opening. Therefore, by competing for binding to the vanilloid site with TRPV1 activators, it exerts a modulatory effect opposite to that of capsaicin (i.e., inhibits the channel, it is a vanilloid antagonist) [146]. In other words, while retaining a relatively large affinity for TRPV1, capsazepine does not elicit the structural response leading to channel opening. While this lack of activity as activator is of clear pharmacological relevance —antagonizing the effect of endogenous vanilloids is one of the putative strategies to achieve analgesia —its molecular determinants remain to be understood and thus we are still far from a rational design of antagonists.

Indeed, despite the fact that single particle cryo electron microscopy has unveiled the three-dimensional structures of the channel in the apo form and in complex with capsaicin, RTX and capsazepine, the electron density maps for these structures did not have initially enough information to unambiguously determine the position of structural waters and to unambiguously determine the conformation of all the side chains of the residues lining the vanilloid pocket. Nevertheless, this structural information paved the way for subsequent computational docking and molecular dynamics studies in which the comparison between different agonists and between agonists and antagonists has been used to rationalize the different behavior of the ligands. These structural studies are opening up a new era for pain killer design in which the accurate knowledge of the specific binding modes gives insights into the design principles of agonists and antagonists [147].

#### 4.3.2. Binding Mode of Capsaicin and RTX

The initial electron density determined by Cao et al. [139] did not possess a resolution large enough to unambiguously determine the spatial location and orientation of capsaicin and RTX. This lack of information motivated a series of computational and joint computational/experimental studies that not only resulted in a more detailed structural picture of vanilloid binding to TRPV1, but also allowed to formulate informed hypotheses about their mode of action. In this regard, one of the most important milestones has been the work of Yang et al. [148], which has shown that capsaicin binds “head down” (with the vanillyl group pointing toward the intracellular milieu) and that the most relevant interactions between the ligand and the channel involve the amide group of capsaicin and the side chain of Thr550 and the hydroxyl from the vanilloid group and the side chain of Glu570. The relevance of these ligand channel interactions was quantitatively assessed via double mutant cycle analysis [149].

A subsequent molecular dynamics investigation by Darre and Domene [150] provided additional elements of support to the notion that that the amide group of capsaicin plays a crucial role in ligand binding: molecular dynamics trajectories and free energy calculations showed that Thr550 and Tyr511 engage in hydrogen bonding interactions, respectively, with the amine and carbonyl groups of capsaicin through bridging water molecules. These water-mediated interactions were confirmed by an independent study by Elokely et al. [38] in which a statistical physics based model was used to find binding regions for tightly bound water molecules. In addition to these polar interactions, this study investigated the role of hydrophobic interactions involving residues from S4 and the linker domain. In particular five residues were predicted and shown experimentally to affect significantly binding upon mutation (Leu515, Leu553, Tyr554, Ile573 and Phe587), thereby providing a stringent test for the structural model. The emerging picture is a tight fit of the vanilloid group in a hydrophobic pocket formed mainly by residues from the C-term region of S4 and the linker. This set of interactions was shown to be crucial for both capsaicin and RTX. Importantly, the largest effect was observed for Leu553 and Tyr554 from S4 and Ile573 from the linker domain.

#### 4.3.3. Mechanism of Activation by Vanilloids

**Figure 2:**
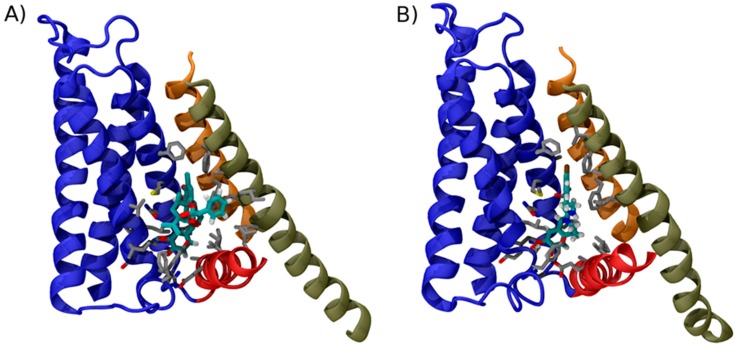
Binding mode of TRPV1 modulators. Structure of TRPV1 in complex with: RTX (**A**); and capsazepine **(B**). For clarity only the structural element surrounding the vanilloid binding site are shown in cartoon representation using the same color code used in Figure 1. Amino acid side chains contacting the ligands are shown as sticks; to highlight the location of ligand in the two structures, the carbon atoms of RTX and capsezepine are highlighted by the cyan color. Note how the conformations of RTX and capsazepine are very similar, except for a phenyl group, which, in RTX, contacts the side chains of S6.

The computational studies based on docking and molecular dynamics highlighted a small set of persistent ligand-channel interactions, which were subsequently experimentally tested. This information enabled to formulate hypotheses on the molecular mechanism of vanilloids and provided a rationale for the novel structural information that became recently available. Indeed, Gao et al. [144] determined the structure of TRPV1 in the apo form and in complex with RTX and capsazepine in a lipid nanodisc environment (Figure 2). The use of this supermolecular assembly, which faithfully reproduces the native lipid bilayer environment of the channel, enabled the authors to obtain extremely well defined electron densities and thus to identify the location and orientation of the ligands with remarkable accuracy. This study confirmed the theoretical prediction that RTX interacts one side with the S1–S4 domain (with residues Ser512, Arg557, Leu515, Val518, and Met547 as well as Leu669, and Thr550) and on the other side with linker residue Ile573 (Figure 2A). Importantly, this observation brings support to the hypothesis raised in the previous computational studies [38,148,150] that the role of agonists is to act as “molecular glue” between the linker and the S1–S4 domain. Importantly, the cryoEM structures provided evidence of a feature deemed crucial for the agonist effect in Yang et al. 2015 [148], i.e., the fact that the vanilloid head group catalyzes the formation of a salt bridge between Glu570 and Arg577, an interaction that contributes to make the S1–S4 and the linker domains a single rigid unit. Importantly, the structure of TRPV1 in complex with capsazepine revealed no salt bridge between these two side chains and, in general, less extensive interactions with the linker domain (Figure 2B).

The overall emerging picture confirms the “pull-and-contact” model proposed initially by Yang et al. [148] in which the binding of vanilloids, by acting as “molecular glue”, promotes the lateral movement of the linker toward the S1–S4 domain. As shown previously for voltage gated ion channels, this conformational transition of the linker domain is able to release the steric hindrance that prevents the splay of the S6 bundle. In other words, in the resting state the four linker domains surround the S6 four-helix bundle lining the pore and act as a “cuff”. Once the linker domains are displaced, the constriction is removed and the S6 helix bundle is free to expand [151]. Strikingly, two independent studies have provided compelling confirmation of this model by engineering RTX sensitivity into TRPV2—a channel that is natively insensitive to this vanilloid. In the first study, Yang et al. [152] introduced the residues Ser512, Phe543, Thr550 and Glu570 (TRPV1 numbering) in the corresponding positions of TRPV2. The resulting construct was shown to be activated by RTX (although the ligand-induced open state was relatively unstable). In the second work, Zhang et al. [153] reported completely consistent observations by engineering a quadruple mutant that introduces Ser512, Met547, Thr550 and Glu570 in the background of TRPV2.

#### 4.3.4. Structure Driven Design of Novel Modulators

The accurate structural information available at this point for TRPV1 in complex with agonists and antagonists, together with an experimentally testable—and to some extent verified—microscopic model of the vanilloid action has already prompted several structure-based studies in the last two years and will, no doubt, catalyze an explosion of rational drug design campaigns in the foreseeable future.

The first study entirely based on this newly available information was that of Feng et al. [154]. The authors of this study used docking and molecular dynamics simulations to extract detailed information about the interactions of two groups of ligands known to have agonist or antagonist action. The accurate three-dimensional structures obtained from these calculations were then used to generate a so-called pharmacophore, an abstract representation of the molecular features most relevant for binding and activity. Thanks to this pharmacophore, the authors were able to screen a virtual library of compounds and extract promising hits. In particular two compounds were found experimentally to be relatively potent (two halogenated diaryl-nitro compounds), that were also investigated through docking and molecular dynamics simulations. The two compounds resulted in 98.2% ± 2.7% and 79.9% ± 4.9% inhibition at a concentration of 30 μM, i.e., they antagonized capsaicin (30 nM) in a calcium uptake assay. These compounds also proved to have relatively high affinity with K_i_ values for capsaicin antagonism of 2.60 ± 0.62 μM and 4.50 ± 0.88 μM. Simulations showed that the compounds interacted stably with Tyr511 and Thr550 via hydrogen bonds. Moreover, the ligands established strong hydrophobic interactions with Met514 and Leu547, i.e., some of the side chains previously shown to affect capsaicin affinity upon mutation. Importantly, these relatively potent antagonists, formed strong hydrogen bonds with Arg557, thereby inhibiting the salt bridge with Glu570 found to be crucial for activation by Cao et al. 2016. Consistently, inactive compounds were not observed to form this hydrogen bond with Arg557.

Building on their initial screen Feng et al. [155] further investigated diaryl molecules and screened a library of diarylurea compounds. The rationale for the choice of this class of molecules is the observation, coming from the previous computational studies, of the crucial energetic role played by the interaction between the capsaicin amide group and the side chain of Thr550. The hypothesis was that, given the relevance of this polar interaction, replacing the amide group with urea should increase affinity and thus potency. Using the hydroxyl of Thr550 as anchoring point for the urea, the library developed chemical diversity by changing the substituents of the two aryl-groups. In addition, in this case, docking and molecular dynamics showed that Tyr511, Leu518, Leu547, Thr550, Asn551, Arg557, and Leu670 were the most important side chains for recognition of the ligand by TRPV1.

A second group identified *N*-(3-fluoro-4-methylsulfonamidomethylphenyl)urea as a novel template for TRPV1 antagonists and developed chemical diversity in various regions of this molecule to generate a library to be screened [156,157]. Importantly, when the binding of this compound was analyzed with computational docking, the urea group forms hydrogen bonds with Tyr511, thereby properly orienting the molecule and favoring the interaction between the two flanking phenyl groups and the hydrophobic regions of the binding site. Importantly, all the residues shown to be crucial for capsaicin binding turned out to form stabilizing interactions with the novel molecules. The *N*-benzylmethanesulfonamide group was found to occupy the crevice lined by the side chains of Tyr511, Tyr554, Ile564, and Ile569. Moreover, the oxygen of the sulfonamide group was shown to be engaged in a hydrogen bond with Ser512, the 3-trifluoromethyl group was found to interact with Leu547 and the methyl group of Thr550 and the 4-methylpiperidine ring in was observed to be in contact with Tyr511, Met514, and Leu515.

Overall, these recent studies have provided proof of concept that the detailed structural information that became available greatly simplifies the search for TRPV1 modulators and molecules with potency comparable to capsaicin can be now easily identified. Despite these success stories, predicting the activity of the ligand, i.e., the agonist vs. antagonist action, remains challenging on purely structural basis. To make progress in this issue additional studies will be needed to develop a quantitative microscopic model that explains activation of TRPV1 by vanilloids.
